# New Liver MR Imaging Hallmarks for Small Hepatocellular Carcinoma Screening and Diagnosing in High-Risk Patients

**DOI:** 10.3389/fonc.2022.812832

**Published:** 2022-03-09

**Authors:** Feifei Gao, Yi Wei, Tong Zhang, Hanyu Jiang, Qian Li, Yuan Yuan, Shan Yao, Zheng Ye, Shang Wan, Xiaocheng Wei, Lisha Nie, Hehan Tang, Bin Song

**Affiliations:** ^1^ Department of Radiology, West China Hospital, Sichuan University, Chengdu, China; ^2^ MR Research China, GE Healthcare, Beijing, China; ^3^ Department of Radiology, Sanya People’s Hospital, Sanya, China

**Keywords:** hepatocellular carcinoma, detection, diagnosis, criteria, magnetic resonance imaging

## Abstract

**Objective:**

Early detection and diagnosis of hepatocellular carcinoma (HCC) is essential for prognosis; however, the imaging hallmarks for tumor detection and diagnosis has remained the same for years despite the use of many new immerging imaging methods. This study aimed to evaluate the detection performance of hepatic nodules in high risk patients using either hepatobiliary specific contrast (HBSC) agent or extracellular contrast agent (ECA), and further to compare the diagnostic performances for hepatocellular carcinoma (HCC) using different diagnostic criteria with the histopathological results as reference standard.

**Methods:**

This prospective study included 247 nodules in 222 patients (mean age, 53.32 ± 10.84 years; range, 22–79 years). The detection performance and imaging features of each nodule were evaluated in all MR sequences by three experienced abdominal radiologists. The detection performance of each nodule on all MR sequences were compared and further the diagnostic performance of various diagnostic criteria were evaluated.

**Results:**

For those patients who underwent ECA-MRI, the conventional imaging hallmark of “AP + PVP and/or DP” was recommended, as 60.19% diagnostic sensitivity, 80.95% specificity and 100% lesion detection rate. Additionally, for those patients who underwent HBSC-MRI, the diagnostic criteria of “DWI + HBP” was recommended. This diagnostic criteria demonstrated, both in all tumor size and for nodules ≤2 cm, higher sensitivity (93.07 and 90.16%, all p <0.05, respectively) and slightly lower specificity (64.71 and 87.50%, all p >0.05, respectively) than that of the European Association for the Study of the Liver (EASL) criteria.

**Conclusions:**

Different abbreviated MR protocols were recommended for patients using either ECA or HBSC. These provided imaging settings demonstrated high lesion detection rate and diagnostic performance for HCC.

## Introduction

Current guidelines recommend every 6 months surveillance by tumor marker measurement and ultrasonography (US) in patients with chronic liver disease (CHD) or liver cirrhosis (1–4). The standard procedure in those situations with elevated level of alpha-fetoprotein (AFP) or detected nodules requires further contrast enhanced ultrasonography (CEUS), dynamic computed tomography (CT), dynamic magnetic resonance imaging (MRI) or even liver biopsy identification (5–7). Multiparametric MRI has been shown to improve the detection of clinically significant hepatic nodules especially in small and early hepatocellular carcinoma (HCC), and evidence also suggested that MRI helps to avoid the detection of clinically insignificant nodules compared with US and CT and avoid unnecessary liver biopsy identification (8–11). As clinical pathways that use liver MR imaging in patients with either elevated serum AFP level or detected hepatic nodules appeared advantageous, the demand for liver MR imaging is increasing.

At present, the multiparametric liver MR imaging has been designed in several different clinical scenarios and thus to answer several different questions such as the presence or absence of liver cancer, characterization of tumor, whether the tumor emboli invaded the portal vein, or with metastatic invasion, and even the presence of microvascular invasion, the tumor histological grade or future remnant liver function estimation (12–18). Of all these clinical scenarios, the liver MR protocols are designed not only emphasized on the diagnosis but further toward a better comprehensive management of treatment and prognostic evaluation (19, 20). In order to satisfy the broad range of clinical needs fulfilled by liver MR imaging, the protocols are designed comprehensively so that multi-planner high-resolution MR sequences and functional MR sequences were included (21, 22). However, these multiparametric liver MR imaging protocols are time consuming and directly influence the patient throughput (23). Additionally, the duration of scanning time is also a major determinant of the direct costs of an MR examination. From the perspective of health economics, the long examination time related patient throughput and direct costs will greatly limit the patient availability of an imaging test. Furthermore, due to more frequent breath-holding times, especially performed with liver MR imaging (24, 25), may further limit the patient acceptance of this imaging test.

Since the multiparametric liver MR imaging protocols are designed in different clinical scenarios and used to deal with different clinical questions, in a man with CHD or liver cirrhosis, the primary concern is whether clinically significant high-risk hepatic nodule is present or not. It has been reported that the abbreviated MRI (aMRI) protocols are applicable in breast cancer and prostate cancer screening which demonstrated high detection and diagnostic sensitivity (23, 26). Thus, with the use of aMRI protocols, it is expected that the problems, namely, patient throughput and high cost caused by long examination time can be solved and further increase the availability of this imaging test. Several published studies have investigated the utilization of aMRI in liver MR imaging. However, for these studies, the nodule size was not strictly restricted (<3 cm) which may potentially increase the screening performance (27). Moreover, previous studies only investigated the use of aMRI in hepatobiliary specific contrast (HBSC) agent (28, 29), but there are still large proportion of extracellular contrast agent (ECA) use. Thus, there is little known about the screening performance of aMRI protocol in the ECA group.

Therefore, the purpose of this study was to prospectively evaluate the detection performance of hepatic nodules in high risk patients using either HBSC or ECA, and to further compare the diagnostic performances for hepatocellular carcinoma (HCC) using different diagnostic criteria with the histopathological results as reference standard.

## Patients and Methods

### Patients

**Figure 1:**
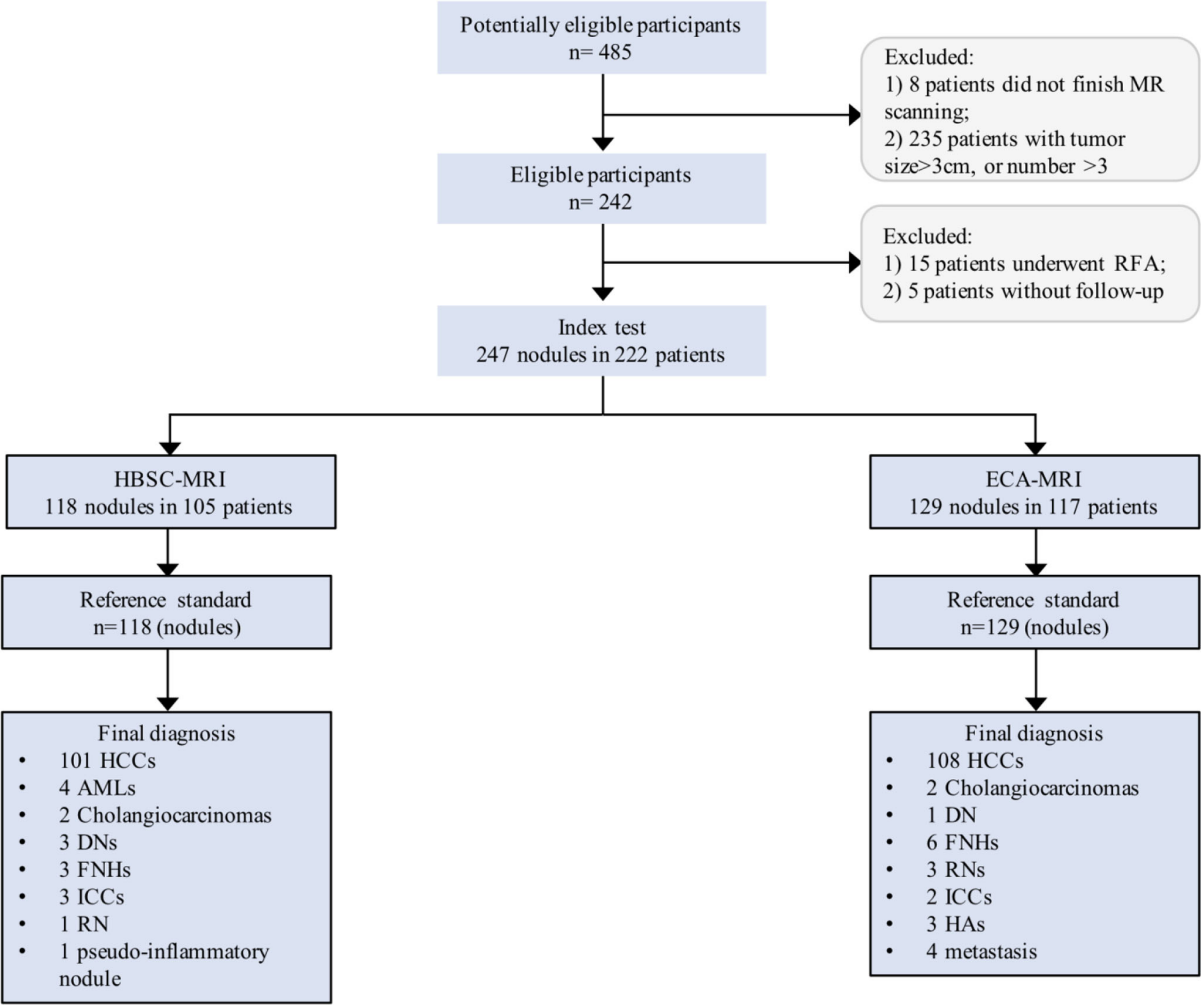
Flowchart of the study population. HCC, hepatocellular carcinoma; AML, angiomyolipoma; DN, dysplastic nodule; FNH, focal nodular hyperplasia; ICC, intrahepatic cholangiocarcinoma; RN, regenerative nodule.

This study was approved by the institutional review board and the written informed consent was obtained from all patients. From January 2017 to June 2020, four hundred and eighty-five consecutive high-risk patients (hepatitis B or C virus infection, or liver cirrhosis) who were suspected of having focal hepatic nodules were potentially enrolled. Of those patients, they were categorized as HBSC and ECA groups according to the use of contrast agent. Patients were ineligible if they have a history of hepatectomy, transarterial chemotherapy (TACE), radiofrequency ablation (RFA), colorectal cancer liver metastasis (CRLM) and MR contraindications. Moreover, the nodule size >3 cm or nodule number >3 in a single patient were also excluded as the aim was to detect the early-stage disease that meet the Barcelona Clinic Liver Cancer (BCLC) staging system. The inclusion criteria were as follows: (1) patients were eighteen years older, (2) nodules were not typically cysts or hemangiomas, and (3) nodules were pathologically confirmed. An experienced coordinator with five years of liver MR imaging retrieved and de-identified all patient images, and hepatic observations presented on MR images were blindly matched with these hepatic nodules presented on surgical removed specimens and with determined histopathological results. Detailed information about the inclusion criteria are shown in **Figure 1**.

### Imaging Technique

MR imaging was carried out using 3.0 T MR systems (Discovery 750w, GE Healthcare, Milwaukee, USA; Skyra 3.0 T, Siemens Healthcare, Erlangen, Germany). Sixteen-channel phased-array torso coils were used for all measurements. In each patient, they were asked to prepare to fast for 6–8 h before MR examination. In addition to localizers, the standard liver MR protocols were: 1) Coronal single shot fast spin echo (SSFSE) T2-weighted imaging, 2) Axial SSFSE T2-weighted with fat saturation (FS), 3) Axial diffusion weighted imaging (DWI), 4) Axial in and out of phase T1-weighted imaging, 5) Axial non-enhanced T1-weighted imaging with FS, 6) Axial contrast-enhanced T1-weighted imaging with FS at arterial phase (AP) (25–30s), portal venous phase (PVP) (60s) and delayed phase (DP) (180s), 7) Coronal contrast-enhanced T1-weighted imaging with FS at delayed phase, and 8) for the HBSC group, the axial contrast-enhanced T1 weighted with FS at transitional phase (3–5 min) and hepatobiliary phase (HBP) (20 min) was also obtained. Contrast agent was administered intravenously using a bolus injection of 0.025 mmol/kg (Primovist, Bayer Pharma AG, Berlin, Germany) at the injection rate of 1 ml/s or for a dose of 0.1 mmol/kg (Magnevist, Bayer Pharma AG, Berlin, Germany) at the injection rate of 2 ml/s, followed by a 20-ml saline flush.

### Image Analysis

Three independent reviewers (readers with 6, 8, and 12 years in liver MR imaging) who were blinded to the histopathological results, clinical and prior imaging data reviewed the MR images; when the three reviewers cannot fully agree with each other, a consensual results was achieved by using the majority assessment results. Firstly, the three reviewers should independently review and determine the presented hepatic nodules on each MR sequence and the detection performance of each MR sequence was calculated. According to the detection performance, different MR sequences were combined as aMRI protocols in different clinical settings. In this study, for the HBSC-MRI group, five aMRI protocols were created, namely,: 1) a-MRI-I: DWI + T2WI; 2) a-MRI-II: DWI + HBP; 3) a-MRI-III: AP+PVP and/or TP+ and/or HBP (Korean Guidelines 2018) (5); 4) a-MRI-IV: AP + PVP and/or +HBP (Japan Society of Hepatology Guideline 2014) (30); 5) a-MRI-V: AP + PVP only (European Association for the Study of the Liver (EASL)) (31); 6) a-MRI-VI: The Liver Imaging Reporting And Data System 2018 (LI-RADS v2018), and when the nodule was assessed as LI-RADS 4 or 5, the nodule was finally determined as HCC with the LI-RADS criteria. In addition, for the ECA-MRI group, three protocols, namely, the conventional imaging hallmark of “AP + PVP and/or DP”, “DWI + T2WI”and LI-RADS v2018 were created. Of these aMRI protocols, the detection performance was calculated and the best aMRI protocol in different clinical settings was determined. Furthermore, the positive imaging features (32, 33) were defined as T2WI: moderate to hyperintensity; DWI: diffusion restriction; HBP: hypointensity but exclude the targetoid sign; Arterial phase hyperenhancement (APHE): the enhancement greater in whole or in part than liver but exclude the rim-like enhancement; and the washout feature at PVP or DP. MR images were presented in a randomized manner, and review of the different aMRI protocols in the same patient was separated by a delay period of 3–4 weeks to minimize recall.

### Statistical Analysis

Lesion detection was summarized by using frequencies and percentages by MR sequence. Of the detection performance by each MR protocol, the per-lesion detection performance was calculated and the detection performance in different MR sequence was compared by using *McNemar’s* test. The diagnostic sensitivity, specificity, negative predictive value (NPV), positive predictive value (PPV), and diagnostic accuracy of ECA-MRI and HBA-MRI were calculated, and also their 95% confidence interval (CI). The comparison of the sensitivity and specificity was tested by using *McNemar’s* test, and the area under curve (AUC) value for each aMRI protocol was calculated. Subgroup analyses were conducted for small (≤20 mm) and larger HCCs (21–30 mm). A two-sided *p*-value of less than 0.05 was considered to indicate statistical significance. All statistical analyses were performed by using a statistical software package [SPSS 23.0 (SPSS Inc., Chicago, IL, USA)].

## Results

### Patients Characteristics

**Figure 2:**
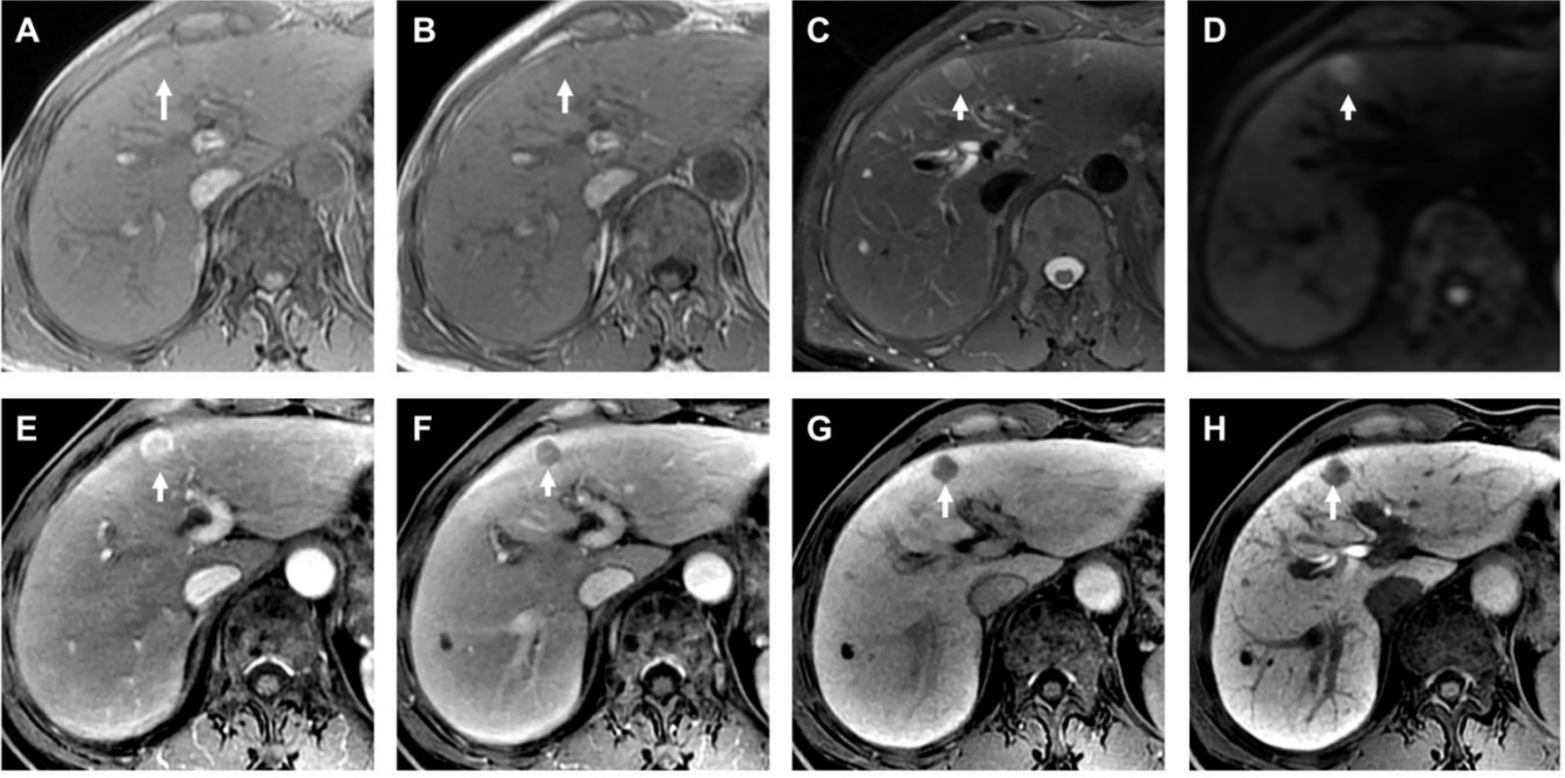
Histopathological confirmed HCC with the size of 1.2 cm. The HBSC-MRI showed an isointense nodule in T1-weighted images in-phase [arrow in **(A)**] and out-phase [arrow in **(B)**] in liver segment IV. This focal liver lesion demonstrated slightly hyperintensity in the T2-weighted image [arrow in **(C)**], and with diffusion restriction [arrow in **(D)**]. The nodule showed typically imaging feature of “wash-in” in the arterial phase **(E)**, and “wash-out” in the portal venous phase **(F)**. The transitional phase [arrow in **(G)**] and hepatobiliary phase [arrow in **(H)**] clearly showed the nodule hypointensity. The final MRI diagnosis was a HCC, confirmed by histology after resection.

**Figure 3:**
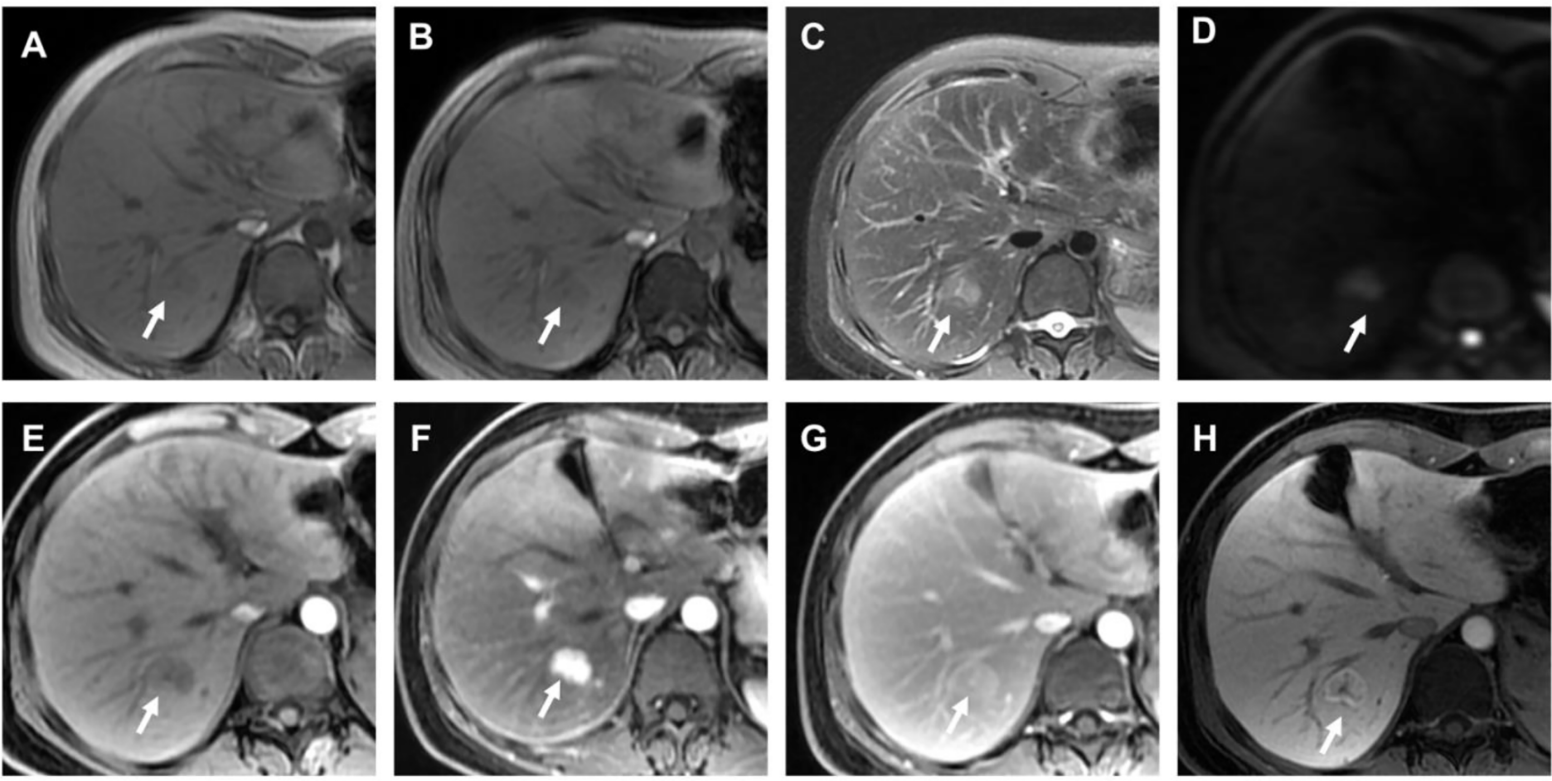
Histopathological confirmed FNH with the size of 2.7 cm. The HBSC-MRI showed an isointense to slightly hypointensity nodule in T1-weighted images in-phase [arrow in **(A)**] and out-phase [arrow in **(B)**] in liver segment VII. This focal liver lesion demonstrated slightly hyperintensity in the T2-weighted image [arrow in **(C)**], and with diffusion restriction [arrow in **(D)**]. The nodule showed hypointense on pre-contrast images **(E)**, strongly hyperenhancement in the arterial phase **(F)**, and isointense compared with normal liver parenchyma in the portal venous phase **(G)**. The hepatobiliary phase [arrow in **(H)**] clearly showed the nodule hyperintensity. The final MRI diagnosis of different criteria (excluding “DWI + T2WI”) was a FNH, confirmed by histology after resection.

In total, there were 247 nodules (mean size, 17.65 ± 6.76 mm, range, 4–30 mm) were analyzed in 222 patients (mean age, 53.32 ± 10.84 years; range, 22–79 years), of whom 170 were men (53.08 ± 10.68 years; range, 22–79 years) and 52 were women (54.12 ± 11.39 years; range, 30–72 years). There were 186 patients with hepatitis B virus infection, and 5 patients with hepatitis C virus infection. Additionally, 219 patients were Child–Pugh A and 3 patients were Child–Pugh B. Of these patients, 200 patients (90.09%; 200/222) had one nodule and 22 patients (9.91%; 22/222) had two or three nodules. The 247 nodules consisted of 209 HCCs (**Figure 2**) and 38 non-HCC nodules (**Figure 3**), all these hepatic nodules were confirmed by histopathological results. In addition, for the HBSC-MRI group, there were 105 patients enrolled; and for the ECA-MRI group, there were 117 patients enrolled.

### Performance of HBSC-MRI

#### Detection Performance of HBSC-MRI

The detailed information about the detection performance of various MR sequences with HBSC-MRI are listed in **Table 1**. The detection performance of HBP was significantly higher than that of DWI (*p* = 0.008), PVP (*p <*0.001) and TP (*p* = 0.016), but no difference was obtained from DWI *vs* PVP (*p* = 0.167), DWI *vs* TP (*p* = 1.000). For the detection performance of aMRI protocols, the a-MRI-II, a-MRI-III and a-MRI-IV detected all the hepatic lesions with the detection performance of 100% (118/118), and followed with a-MRI-I (94.07%, 111/118), a-MRI-V and a-MRI-VI (93.22%, 110/118).

**Table 1:** Detection performance of the MR sequence and aMRI protocols with HBSC-MRI.

Variable	Total lesions (n = 118)		Detection performance (100%)	lesion size, n (100%)		
	No. of detected lesions	No. of undetected lesions		≤1.0 (cm), n = 28	1.1–2.0 (cm), n = 41	2.1–3.0 (cm), n = 49
T1WI	82	36	69.49 (82/118)	21.43 (6/28)	70.73 (29/41)	95.92 (47/49)
T2WI	102	16	86.44 (102/118)	71.43 (20/28)	82.93 (34/41)	97.96(48/49)
DWI	110	8	93.22 (110/118)	89.29 (25/28)	87.80 (36/41)	100 (49/49)
Pre	82	36	69.49 (82/118)	39.29 (11/28)	63.41 (26/41)	91.84 (45/49)
AP	86	32	72.88 (86/118)	64.29 (18/28)	73.17 (30/41)	77.55 (38/49)
PVP	103	15	87.29 (103/118)	75 (21/28)	87.80 (36/41)	93.88 (46/49)
DP/TP	111	7	94.07 (111/118)	89.29 (25/28)	92.68 (38/41)	97.96 (48/49)
HBP	118	0	100 (118/118)	100 (28/28)	100 (41/41)	100 (49/49)
aMRI-I	111	7	94.07 (111/118)	89.29 (25/28)	90.24 (37/41)	100 (49/49)
aMRI-II	118	0	100 (118/118)	100 (28/28)	100 (41/41)	100 (49/49)
aMRI-III	118	0	100 (118/118)	100 (28/28)	100 (41/41)	100 (49/49)
aMRI-IV	118	0	100 (118/118)	100 (28/28)	100 (41/41)	100 (49/49)
aMRI-V	110	8	93.22 (110/118)	89.29 (25/28)	92.68 (38/41)	95.92 (47/49)
aMRI-VI	110	8	93.22 (110/118)	89.29 (25/28)	92.68 (38/41)	95.92 (47/49)

T1WI, T1-weighted imaging; T2WI, T2-weighted imaging; DWI, diffusion weighted imaging; AP, arterial phase; PVP, portal venous phase; DP, delayed phase; TP, transitional phase; HBP, hepatobiliary phase.

#### Diagnostic Performance of HBSC-MRI

**Figure 4:**
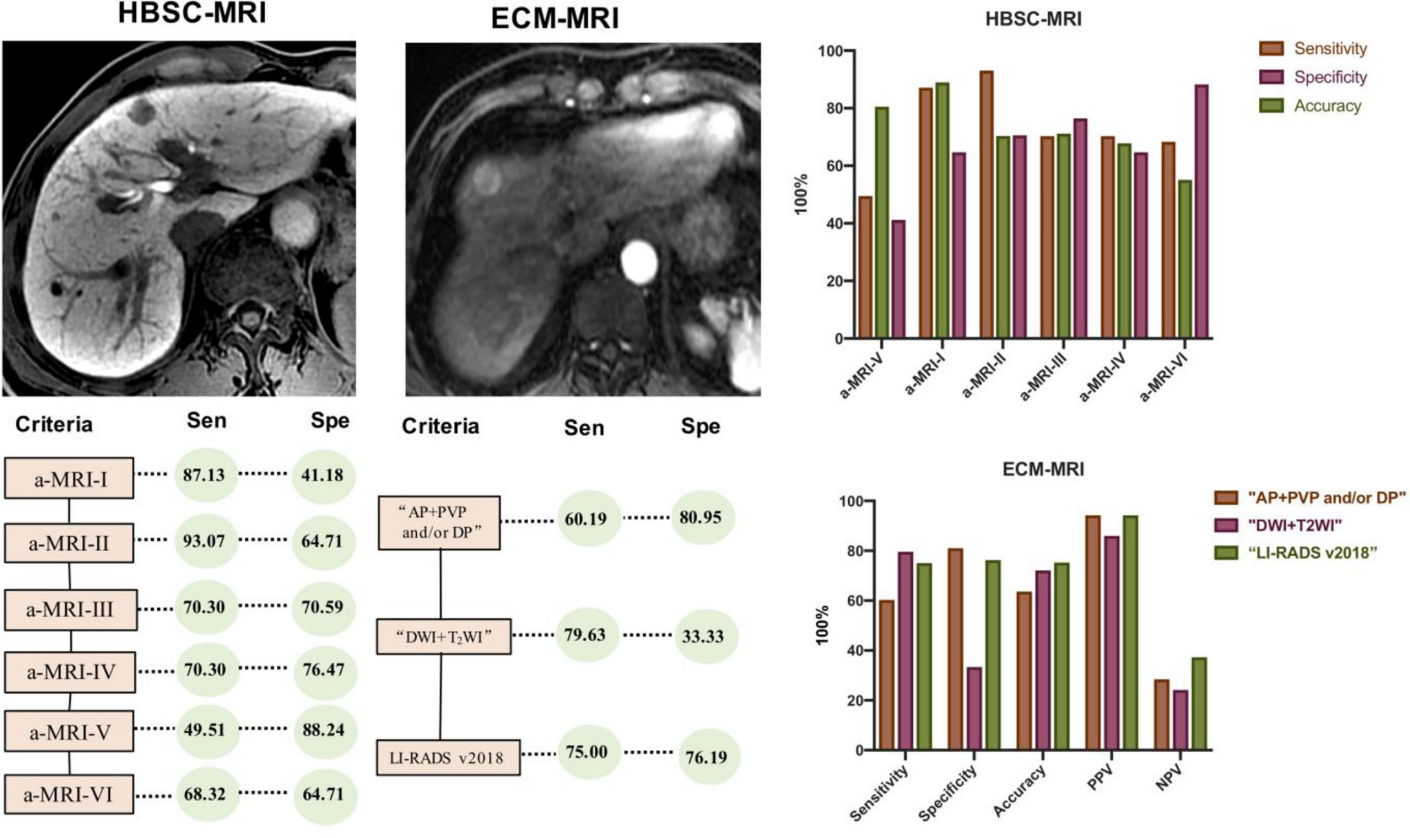
Comparison of the diagnostic sensitivity and specificity of various diagnostic criteria in diagnosing HCC.

Using these criteria (**Table 2** and **Figure 4**), the highest diagnostic sensitivity of 93.07% (94/101) was achieved by using a-MRI-II, and followed with aMRI-I (87.13%, 88/101), Korean Guideline 2018 (70.30%, 71/101), Japan Society of Hepatology Guideline 2014 (70.30%, 71/101), LI-RADS 2018 (68.32%, 69/101) and which were significantly higher than that of EASL Guideline with the sensitivity of 49.50% (50/101) (all *p <*0.05). For the diagnostic specificity comparison, the EASL Guideline showed the highest diagnostic specificity of 88.24% (15/17) and which was significant higher than that of aMRI-I (41.18%, 7/17) (*p* = 0.021), but no significant difference was obtained when compared with that of aMRI-II (64.71%, 11/17), Korean Guidelines 2018 (a-MRI-III) (70.59%, 12/17), Japan Society of Hepatology Guideline 2014 (a-MRI-IV) (76.47%, 13/17) and LI-RADS v2018 (a-MRI-VI) (64.71%, 11/17) (all *p >*0.05). The AUC values for aMRI-I, aMRI-II, Korean Guidelines 2018, Japan Society of Hepatology Guideline 2014, EASL Guideline (a-MRI-V), and LI-RADS v2018 were 0.789, 0.704, 0.734, 0.689, 0.642 and 0.665, respectively.

**Table 2:** Diagnostic performance of these criteria using HBSC-MRI.

Imaging set	Se (95% CI) %	Sp (95%CI) %	Ac (95%CI) %	PPV (95%CI) %	NPV (95%CI) %	Youden index	AUC
EASL Guideline	49.50 (39.59–59.42)	88.24 (66.67–95.00)	55.09 (45.98–64.19)	96.15 (85.75–98.56)	22.73 (12.35–33.11)	0.38	0.642 (0.485–0.798)
	…	…	…	…	…		
DWI + T2WI	87.13 (80.48–93.77)	41.18 (15.09–67.26)	80.51 (73.26–87.76)	89.80 (83.7–95.9)	35.00 (12.1–57.9)	0.28	0.789 (0.648–0.93)
	<0.001	0.021					
DWI + HBP	93.07 (88.03–98.11)	64.71 (39.38–90.03)	88.98 (83.25–94.72)	94.00 (89.26–98.74)	61.11 (36.17–86.06)	0.58	0.704 (0.569–0.84)
	<0.001	0.125					
Korean Guidelines 2018	70.30 (61.23–79.36)	70.59 (46.44–97.74)	70.34 (61.98–78.70)	93.42 (87.72–99.12)	28.57 (14.32–42.82)	0.41	0.734 (0.606–0.862)
	<0.001	0.250					
Japan Society of Hepatology Guideline 2014	70.30 (61.23–79.36)	76.47 (53.99–98.95)	71.19 (62.89–79.48)	94.67 (89.46–99.87)	30.23 (15.93–44.93)	0.47	0.689 (0.569–0.809)
	<0.001	0.500					
LI-RADS v2018	68.32 (59.09–77.55)	64.71 (39.38–90.03)	67.80 (59.24–76.35)	92.00 (85.72–98.28)	25.58 (11.99–39.17)	0.33	0.665 (0.523–0.807)
	<0.001	0.125					

EASL, European Association for the Study of the Liver; LI-RADS v2018, Liver Imaging Reporting And Data System version 2018.

Regarding the performance of tumor size ≤20 mm (**Table 3**), the a-MRI-II achieved the highest diagnostic sensitivity and specificity of 90.16 and 87.50%, and the Youden index was 0.78. Considering the classic imaging hallmarks (EASL Guideline), the sensitivity and specificity were 44.26 and 87.50%, respectively. The diagnostic sensitivity of the EASL Guideline was significantly lower than other imaging criteria (all *p <*0.05), but no significant difference was obtained from the diagnostic specificity (all *p >*0.05). For the tumor size of 20-30 mm (**Table 3**), the EASL Guideline achieved the highest diagnostic specificity, which was significant higher than a-MRI-I (*p* = 0.031), but no difference was obtained from other criteria (all *p >*0.05).

**Table 3:** Diagnostic performance of these criteria using HBSC-MRI with different tumor size.

Imaging set	Nodule size ≤20 mm, n = 69			Youden index	Nodule size 21–30 mm, n = 49			Youden index
	Se (95% CI) %	Sp (95% CI) %	Ac (95% CI) %		Se (95% CI) %	Sp (95% CI) %	Ac (95% CI) %	
EASL Guideline	44.26 (31.82–56.34)	87.50 (66.67–92.86)	49.28 (37.18–61.37)	0.32	57.50 (42.50–72.50)	88.89 (72.73–93.60)	63.27 (49.27–77.26)	0.46
	…	…			…	…		
DWI+T2WI	78.69 (67.21–87.30)	62.50 (20.00–87.50)	76.81 (66.60–87.02)	0.41	100 (91.24–100)	22.22 (6.3–54.75)	85.71 (75.56–95.87)	0.22
	<0.001	0.625			<0.001	0.031		
DWI+HBP	90.16 (80.42–95.44)	87.50 (66.67–92.86)	89.86 (82.55–97.16)	0.78	97.50 (92.31–97.74)	44.44 (16.67–77.78)	87.76 (78.24–97.27)	0.42
	<0.001	1.000			<0.001	0.125		
Korean Guidelines 2018	68.85 (56.67–78.95)	75.00 (33.30–90.00)	69.57 (58.43–80.70)	0.44	72.50 (56.40–84.62)	66.67 (25.00–88.89)	71.43 (58.32–84.54)	0.39
	<0.001	1.000			0.031	0.500		
Japan Society of Hepatology Guideline 2014	68.85 (56.67–78.95)	87.50 (66.67–92.86)	71.01 (60.04–81.99)	0.44	72.50 (56.40–84.62)	66.67 (25.00–88.89)	71.43 (58.32–84.54)	0.39
	<0.001	1.000			0.031	0.500		
LI-RADS v2018	52.46 (39.56–65.36)	87.50 (66.67–92.86)	56.52 (44.53–68.52)	0.40	92.50 (80.97–98.03)	44.44 (16.67–77.78)	83.67 (72.95–94.40)	0.37
	<0.001	1.000			<0.001	0.125		

T2-weighted imaging; DWI, diffusion weighted imaging; HBP, hepatobiliary phase; EASL, European Association for the Study of the Liver; LI-RADS v2018, Liver Imaging Reporting And Data System version 2018.

### Performance of ECA-MRI

#### Detection Performance of ECA-MRI

The detailed information about the detection performance of various MR sequences with ECA-MRI are listed in **Table 4**. The detection performance of PVP and DP were significantly higher than that of T1WI (*p <*0.001), T2WI (*p <*0.001) and DWI (*p* = 0.049), but no difference was obtained from AP (*p* = 1.000). For the combination use of MR sequence, the “T2WI + DWI” detected 123 (95.35%, 123/129) lesions, and “AP + PVP and/or DP” and LI-RADS v2018 detected all the 129 (100%, 129/129) lesions, and no difference was obtained from the detection performance of these combined sequences (*p* = 1.000).

**Table 4:** Detection performance of the MR sequence and aMRI protocols with ECA-MRI.

Variable	Total lesions (n = 129)		Detection performance (100%)	lesion size, n (100%)		
	No. of detected lesions	No. of undetected lesions		≤1.0 (cm), n = 20	1.1–2.0 (cm), n = 69	2.1–3.0(cm), n = 40
T1WI	99	30	76.74 (99/129)	65 (13/20)	76.81 (53/69)	82.50 (33/40)
T2WI	112	17	86.82 (112/129)	75 (15/20)	85.51 (59/69)	95 (38/40)
DWI	113	16	87.60 (113/129)	70 (14/20)	88.41 (61/69)	95 (38/40)
Pre	101	28	78.29 (101/129)	55 (11/20)	79.71 (55/69)	87.50 (35/40)
AP	123	6	95.35 (123/129)	100 (20/20)	92.75 (64/69)	97.50 (39/40)
PVP	122	7	94.57 (122/129)	80 (16/20)	95.65 (66/69)	100 (40/40)
DP	122	7	94.57 (122/129)	80 (16/20)	95.65 (66/69)	100 (40/40)
T2WI + DWI	123	6	95.35 (123/129)	90 (18/20)	95.65 (66/69)	97.50 (39/40)
AP + DP/PVP	129	0	100 (129/129)	100 (20/20)	100 (69/69)	100 (40/40)
LI-RADS v2018	129	0	100 (129/129)	100 (20/20)	100 (69/69)	100 (40/40)

T1WI, T1-weighted imaging; T2WI, T2-weighted imaging; DWI, diffusion weighted imaging; AP, arterial phase; PVP, portal venous phase; DP, delayed phase; LI-RADS v2018, Liver Imaging Reporting And Data System version 2018.

#### Diagnostic Performance of ECA-MRI

Using the conventional imaging hallmark of “AP + PVP and/or DP”, the diagnostic sensitivity was 60.19% (65/108) and which was significantly lower than that of “DWI + T2WI” (79.63%, 86/108) and LI-RADS v2018 (75.00%, 81/108). For the diagnostic specificity comparison, the specificity was 80.95% (17/21) for conventional imaging hallmark of “AP + PVP and/or DP” and which was significantly higher than that of “DWI + T2WI”(33.33%, 7/21), but no difference was obtained when compared with LI-RADS v2018 (76.19%, 16/21). The AUC value for the imaging hallmark of “AP + PVP and/or DP”, “DWI + T2WI” and LI-RADS v2018 was 0.706, 0.565, and 0.756, respectively (**Table 5**).

**Table 5:** Diagnostic performance of these criteria using ECA-MRI with different tumor size.

Variable	Se (95% CI) %	Sp (95% CI) %	Ac (95% CI) %	PPV (95% CI) %	NPV (95% CI) %	Youden index	AUC
**“AP + PVP and/or DP”**							
**All size**	60.19 (50.80–69.57)	80.95 (62.64–99.27)	63.57 (55.15–71.98)	94.20 (88.55–99.86)	28.33 (16.59–40.07)	0.41	0.706
**≤20 mm**	60 (48.40–70.89)	78.57 (50.00–92.86)	62.92 (52.69–73.15)	93.75 (82.17–97.92)	26.83 (14.61–42.51)	0.39	…
**21–30 mm**	60.61 (42.33–75.00)	85.71 (60.00–92.31)	65 (49.55–80.45)	95.24 (85.00–96.30)	31.58 (12.50–55.56)	0.46	…
**“DWI + T2WI”**							
**All size**	79.63 (71.91–87.35)	33.33 (11.35–55.32)	72.09 (64.25–79.94)	86 (79.08–92.92)	24.14 (7.57–40.70)	0.13	0.565
	0.001	0.031					
**≤ 20 mm**	73.33 (63.09–83.58)	35.71 (7.00–64.42)	67.42 (57.49–77.34)	85.94 (77.19–94.69)	20.00 (3.15–36.85)	0.09	…
	0.078	0.146					
**21-30 mm**	93.94 (78.16–97.14)	28.57 (9.09–59.07)	82.5 (70.19–94.81)	86.11 (69.44–94.44)	50 (16.50–75.00)	0.22	…
	0.001	0.219					
**“LI-RADS v2018”**							
**All size**	75.00 (66.70–83.30)	76.19 (56.32–96.06)	75.19 (67.64–82.75)	94.19 (89.14–99.23)	37.21 (22.16–52.26)	0.51	0.756
	<0.001	1.000					
**≤20 mm**	68.00 (57.20–78.80)	78.57 (50.99–98.16)	69.66 (59.92–79.40)	94.44 (85.13–98.76)	31.43 (15.25–47.61)	0.47	…
	0.031	1.000					
**21–30 mm**	90.91 (78.56–96.26)	71.43 (58.32–84.54)	87.50 (76.79–98.21)	93.75 (82.17–97.92)	62.50 (20.00–87.50)	0.22	…
	0.002	1.000					

T2WI, T2-weighted imaging; DWI, diffusion weighted imaging; AP, arterial phase; PVP, portal venous phase; DP, delayed phase; LI-RADS v2018, Liver Imaging Reporting and Data System version 2018.

Regarding the tumor size ≤20 mm, the diagnostic sensitivity for imaging hallmark of “AP + PVP and/or DP” was significantly lower than LI-RADS v2018 (*p* = 0.031), but no difference was obtained from the comparison of specificity (all *p >*0.05). In addition, for the tumor size of 21–30 mm, the diagnostic sensitivity for imaging hallmark of “AP + PVP and/or DP” was significant lower than that of “DWI + T2WI” and LI-RADS v2018 (all *p <*0.05), but no difference was obtained from specificity (all *p >*0.05).

## Discussion

In the present study, the detection performance of various MR sequences in detecting hepatic nodules in high-risk patients were evaluated and further the diagnostic sensitivity and specificity of different clinical guidelines and aMRI protocols using either HBSC-MRI or ECA-MRI were compared. The results showed that HBP was most sensitive in detecting hepatic nodules with HBSC-MRI, while AP was most sensitive for conventional ECA-MRI. Regarding the different clinical guidelines and aMRI protocols in diagnosing HCC, in HBSC-MRI group, the use of “DWI + HBP” as a diagnostic criteria showed the highest diagnostic sensitivity, but the EASL criteria showed the lowest diagnostic sensitivity no matter the size of tumor. Nevertheless, the EASL criteria showed the highest diagnostic specificity in diagnosing HCC. Additionally, in the ECA-MRI group, the diagnostic sensitivity of “DWI + T2WI”as diagnostic criteria was higher than that of the conventional imaging hallmark of “AP + PVP and/or DP”, but the diagnostic specificity was greatly reduced.

Our results found that HBP detected all the hepatic nodules in HBSC-MRI group and followed with TP and DWI, however, for the patients underwent with ECA-MRI, AP showed the highest detection ability and subsequent with PVP/DP and DWI. It is worth noting that compared with HBP, AP alone cannot detect all the hepatic lesions and only when combined with PVP/DP, all the suspected hepatic lesions can be detected with ECA-MRI. Our results were in line with Kim et al. (34) who also found that additional evaluation by MR imaging with gadoxetic acid can led to the detection of additional HCC nodules in 16% of patients. The reason why HBP can detect more hepatic nodules than AP might be explained by that the alteration of hepatic membrane function developed earlier than that of the abnormal hepatic blood supply during the process of carcinogenesis. Another noteworthy issue is that the detection performance of AP with ECA-MRI was obviously higher than that with HBSC-MRI. In this study, AP only detected 72.88% (86/118) of nodules with HBSC-MRI, but about 95.35% (123/129) of hepatic nodules were detected with ECA-MRI, this mainly caused by the high incidence rate of transient severe motion in AP with HBSC-MRI and which may directly reduce the detection performance. Thus, there is still a concern about whether AP is still necessary for these patients underwent with HBSC-MRI for nodule detection.

Previous studies (35–37) have directly compared the different diagnostic criteria for the diagnosis of HCC with HBSC-MRI, Paisant et al. (35) found that when use the histological results as the gold standard, the Korean guideline 2018 showed the highest diagnostic sensitivity of 71.6% compared with that of 67.6% with the Japan Society of Hepatology guideline 2014 and 45.1% of the EASL guideline. Another study conducted by Kim et al. (36) also found that when extended the imaging feature of washout from PVP to HBP, the diagnostic sensitivity increased from 75.3 to 95.2%. Our results also showed that when the diagnostic criteria containing TP/HBP (70.30%), the diagnostic sensitivity has significantly increased compared with the EASL criteria (49.51%). More importantly, our study proposes another more sensitive diagnostic criteria for HCC that is the combined use of “DWI + HBP”, the diagnostic sensitive was 93.07, 90.16, and 97.50% for the tumor size of 0–30 mm, ≤20 mm and 21–30 mm, respectively, and which was significantly higher than that of the EASL and Asian guidelines. This can be explained by the fact that as the detection is the first step of tumor diagnosis, compared with other imaging criteria containing AP which often neglect the hepatic nodules because of the transient severe motion, the “DWI + HBP” can maximally ensure the detection of hepatic nodules and thus further improve the diagnostic performance.

Additionally, excepting for the diagnostic sensitivity, the diagnostic specificity is also essential for clinical options. The EASL criteria keeps the highest diagnostic specificity in all tumor size category with HBSC-MRI, and the specificity of “DWI + HBP” was slightly numerical lower than that of the EASL and Asian criteria, but no statistically difference was obtained. However, for the diagnostic specificity of “DWI + HBP”, the further stratified analysis results showed that the highest specificity was obtained with the tumor size ≤20 mm and which was equal to the EASL criteria. The unsatisfactory diagnostic specificity was obtained with tumor range of 21–30 mm, as two AMLs, two CCs and 1 pseudo-inflammatory tumor was misdiagnosed as the imaging feature often mimics HCC and which is hard to differentiate. Moreover, the highest Youden index value was obtained by using the criteria of “DWI + HBP” which means that by using this criteria, better screening and greater authenticity was achieved.

Our results found that the diagnostic criteria of “DWI + T2WI” showed high diagnostic sensitivity compared with that of “AP + PVP and/or DP” with ECA-MRI, but the diagnostic specificity of “DWI + T2WI” lower than that of “AP + PVP and/or DP”. Considering this situation, for ensuring both diagnostic sensitivity and specificity, we recommend that for patients underwent with ECA-MRI, the conventional imaging hallmark of “AP + PVP and/or DP” was strongly advocated, and for patients only underwent with plain liver MR scanning, the “DWI + T2WI”was recommended because of its high diagnostic sensitivity. In addition, another important question which merit for discussing is that whether the low diagnostic specificity of “DWI + T2WI” means that this diagnostic criteria is of little value in diagnosing HCC. On the contrary, in clinical practice, the T2WI hyperintensity nodule with diffusion restriction just goes to show the possibility of malignant hepatic nodule but not HCC specific, and which can also be used to differentiate RN and DN (8, 35, 36, 38). Thus, the diagnostic criteria of “DWI + T2WI” have good suggestive significance as whether the malignant hepatic nodule is presented, and the further enhanced MR imaging is needed.

This study has several limitations. First, only a limited number of non-HCC hepatic nodules were included in our study, and which may potentially lead to the bias of the diagnostic specificity. However, this reflects the best current clinical practice that most nodules in high-risk patients were HCCs. Second, despite the resected liver specimens and final pathological results were taken as the reference standard; a substantial number of hepatic nodules may be missed as we did not have whole liver pathological evaluation. As one of the purposes of this study was to detect the early-stage disease that meet the BCLC staging system, the partial liver resection is the standard surgery for the enrolled patients. Therefore, the high detection rates in this study represent relative, but not absolute. However, strictly hepatic nodule detection was made by using the resected specimens, and the detected nodules on liver specimens were matched with the MR images. Thus, to a certain extent, the results of high detection rate have certain representation. Third, the head-to-head comparison of the extracellular contrast agents and hepatobiliary contrast agents in the same patient cohort was not conducted as the patients cannot underwent both MR scanning in a short period. Previous study conducted by Paisant et al. (35) has directly compared extracellular and hepatobiliary MR contrast agents for the diagnosis of small HCCs and found that hepatobiliary MR outperforms extracellular contrast agents in diagnosing small HCCs. Thus, large sample, multi-center directly comparison and validation of the two contrast agents are needed in future.

### Conclusions

In conclusion, for patients with HBSC-MRI, our new diagnostic criteria, based on the evaluation of HBP and DWI, demonstrated a higher lesion detection rate, and a significantly higher diagnostic sensitivity compared with that of the EASL and Asian guidelines and a specificity slightly but not significantly lower than that of EASL and Asian guidelines for HCC. Our study also shows that for patients with ECA-MRI, the conventional diagnostic hallmark showed the highest detection and diagnostic performance, suggesting that the application of diagnostic criteria for HCC need to be differentiated in patients using HBSC-MRI and those with ECA-MRI.

## Data Availability Statement

The raw data supporting the conclusions of this article will be made available by the authors, without undue reservation.

## Ethics Statement

The studies involving human participants were reviewed and approved by the West China Hospital, Sichuan University. The patients/participants provided their written informed consent to participate in this study.

## Author Contributions

All authors participated in the interpretation of study results, and in the drafting, critical revision, and approval of the final version of the manuscript. FG YW, HJ, HT, TZ, XW, LN, and BS were involved in the study design. YW, TZ, QL, YY, SW, and ZY were investigators in the study and were involved in the data collection. FG, YW, QL, SY, and SW were involved in the data analysis. FG YW, HT, and BS wrote the manuscript. All authors listed have made a substantial, direct, and intellectual contribution to the work and approved it for publication.

## Funding

This work was supported by the Science and Technology Support Program of Sichuan Province (Grant numbers 2021YFS0144, 2021YFS0021), the China Postdoctoral Science Foundation (2021M692289), and the Post-Doctor Research Project, West China Hospital, Sichuan University (Grant number 2020HXBH130).

## Conflict of Interest

Authors XW and LN were employed by GE Healthcare.

The remaining authors declare that the research was conducted in the absence of any commercial or financial relationships that could be construed as a potential conflict of interest.

## Publisher’s Note

All claims expressed in this article are solely those of the authors and do not necessarily represent those of their affiliated organizations, or those of the publisher, the editors and the reviewers. Any product that may be evaluated in this article, or claim that may be made by its manufacturer, is not guaranteed or endorsed by the publisher.
